# When Imaging Deceives: Recurrent Thromboembolic Strokes From Polymicrobial Bioprosthetic Valve Infection With Repeatedly Negative Transesophageal Echocardiography

**DOI:** 10.7759/cureus.113015

**Published:** 2026-07-20

**Authors:** Sarah Farnand, Sarah AbuKamal, Caroline Pinkerman, Jill Patel, Abdelrahman Mansour

**Affiliations:** 1 Internal Medicine, Midwestern University, Downers Grove, USA; 2 Internal Medicine, Franciscan Health, Chicago, USA

**Keywords:** bioprosthetic valve, duke criteria, enterococcus, prosthetic valve endocarditis, pseudomonas aeruginosa, septic emboli, source control, thromboembolic stroke

## Abstract

Prosthetic valve endocarditis (PVE) is a diagnostically challenging and frequently fatal infection in which echocardiography, the diagnostic cornerstone, is substantially less sensitive than in native valve disease. We describe a 67-year-old man with a bioprosthetic aortic valve who was hospitalized twice within three months, each admission characterized by polymicrobial bacteremia with *Pseudomonas aeruginosa* and *Enterococcus faecalis*, magnetic resonance imaging-confirmed bilateral thromboembolic cerebral infarcts, and septic shock. Transthoracic and transesophageal echocardiography (TTE/TEE) were performed during both hospitalizations and repeatedly failed to demonstrate valvular vegetations. Despite the absence of echocardiographic evidence of endocardial involvement, the clinical constellation of a prosthetic valve, persistent and recurrent bacteremia with biofilm-forming organisms, recurrent septic cerebral emboli, and failure of prolonged targeted antimicrobial therapy to achieve durable bacteremic clearance satisfied the modified Duke criteria for definite infective endocarditis (IE) on both admissions. This case underscores that a negative echocardiogram does not exclude PVE, that the modified Duke criteria enable a definite diagnosis when one major and three minor criteria are met, even when imaging is unrevealing, and that recurrent bacteremia with septic embolization in a patient with prosthetic material constitutes a compelling indication for surgical source control irrespective of echocardiographic findings. After multidisciplinary evaluation, the patient was transferred to a tertiary center for consideration of valve explantation. Clinicians must resist anchoring on false-negative imaging and allow the totality of clinical, microbiological, and radiographic data to guide definitive management.

## Introduction

Infective endocarditis (IE) is a life-threatening infection of the endocardial surface of the heart, most commonly involving the cardiac valves [1]. Its diagnostic hallmark is the vegetation, an infected mass of microorganisms, fibrin, and platelets adherent to the valve. In the modern era of cardiac surgery and valvular intervention, prosthetic valve endocarditis (PVE) represents an increasingly encountered and diagnostically challenging form of the disease, with an incidence of 0.3% to 1.2% per patient-year and mortality approaching 20-40% even with optimal management [2,3].

Echocardiography, both transthoracic (TTE) and transesophageal (TEE), is the cornerstone of IE diagnosis, but its sensitivity for PVE is considerably lower than for native-valve disease. TEE detects vegetations in 87-100% of native-valve IE cases, yet sensitivity falls to approximately 82-96% for PVE and lower still for bioprosthetic valves [3]. The reduced yield reflects acoustic shadowing and reverberation artifact, that is, signal dropout produced by the dense prosthetic material that obscures the adjacent leaflets and periannular tissue, together with the difficulty of resolving small or non-oscillating vegetations against a prosthesis [3].

This diagnostic gap has direct clinical consequences: a clinician who relies solely on echocardiography risks anchoring on a false-negative result, delaying escalation of therapy and, critically, deferring surgical consultation for source control in refractory bacteremia. The modified Duke criteria [4], and their subsequent 2023 Duke-International Society for Cardiovascular Infectious Diseases (Duke-ISCVID) update, were developed to mitigate precisely this limitation by integrating clinical, microbiological, and imaging data so that IE can be diagnosed even when echocardiography is unrevealing.

We present a man with a bioprosthetic aortic valve hospitalized twice within three months, each admission marked by polymicrobial bacteremia with *Pseudomonas aeruginosa* and *Enterococcus faecalis*, both aggressive biofilm-forming organisms that encase themselves in a protective matrix on prosthetic surfaces and resist antibiotic eradication, MRI-confirmed bilateral thromboembolic strokes from septic emboli (fragments of infected vegetation that break off and lodge in cerebral arteries), and septic shock. TTE and TEE failed to visualize vegetations on both admissions. This case addresses a specific and underexplored problem: how to diagnose and manage suspected PVE when both TTE and TEE are repeatedly negative. It illustrates that clinical reasoning anchored in the modified Duke criteria must govern management and that persistent bacteremia without a clear alternative cardiac explanation demands aggressive pursuit of surgical source control regardless of echocardiographic findings.

## Case presentation

Clinical course in brief

Over a three-month period, this patient had two hospitalizations. In the first (January), he presented with altered mental status, was found to have polymicrobial *P. aeruginosa* and *E. faecalis* bacteremia persisting beyond 72 hours with concordant organisms on lower-extremity wound cultures, sustained multifocal embolic strokes, developed septic shock, cleared his bacteremia with antimicrobial therapy, and was discharged to complete outpatient intravenous antibiotics. Approximately six weeks later, during active therapy, he was readmitted (March) with recurrent altered mental status, the identical organism pair, and new bilateral embolic infarcts, at which point the clinical picture fulfilled the modified Duke criteria for definite IE, and he was referred for surgical source control (Table 1).

**Table 1:** Comparison of the two hospitalizations SIRS: systemic inflammatory response syndrome; MICU: medical intensive care unit; AKI: acute kidney injury; PICC: peripherally inserted central catheter; TTE/TEE: transthoracic/transesophageal echocardiography

Feature	First admission (January)	Second admission (~6 weeks later)
Presentation	Acute altered mental status; SIRS	Recurrent acute altered mental status
Hemodynamics	Vasopressor-dependent septic shock (MICU)	Stabilized; no repeat shock
Blood cultures	*P. aeruginosa* + *E. faecalis*	*P. aeruginosa* + *E. faecalis* (identical pair)
Wound cultures	*P. aeruginosa* +* E. faecalis *(concordant; portal of entry)	Not repeated
Duration of bacteremia	Persistent >72 h; cleared before discharge	Recurrent on therapy; cleared with susceptibility-guided therapy
Key laboratory findings	Leukocytosis; pre-renal AKI (vancomycin held)	Leukocytosis; recurrent bacteremia on therapy
Brain MRI	Multifocal bilateral acute/subacute infarcts	New bilateral acute infarcts
TTE / TEE	Both negative for vegetation	Both negative for vegetation
Antimicrobial therapy	Cefepime + linezolid + tobramycin (vancomycin held for AKI); discharged on cefepime ×6 wk + tobramycin ×2 wk (PICC)	Meropenem + linezolid, de-escalated to piperacillin-tazobactam + tobramycin + oral ciprofloxacin per susceptibilities
Anticoagulation	Apixaban →heparin → warfarin	Continued warfarin
Disposition	Home with PICC for outpatient IV therapy	Transfer to a tertiary center for surgical evaluation

First Admission (January)

**Figure 1:**
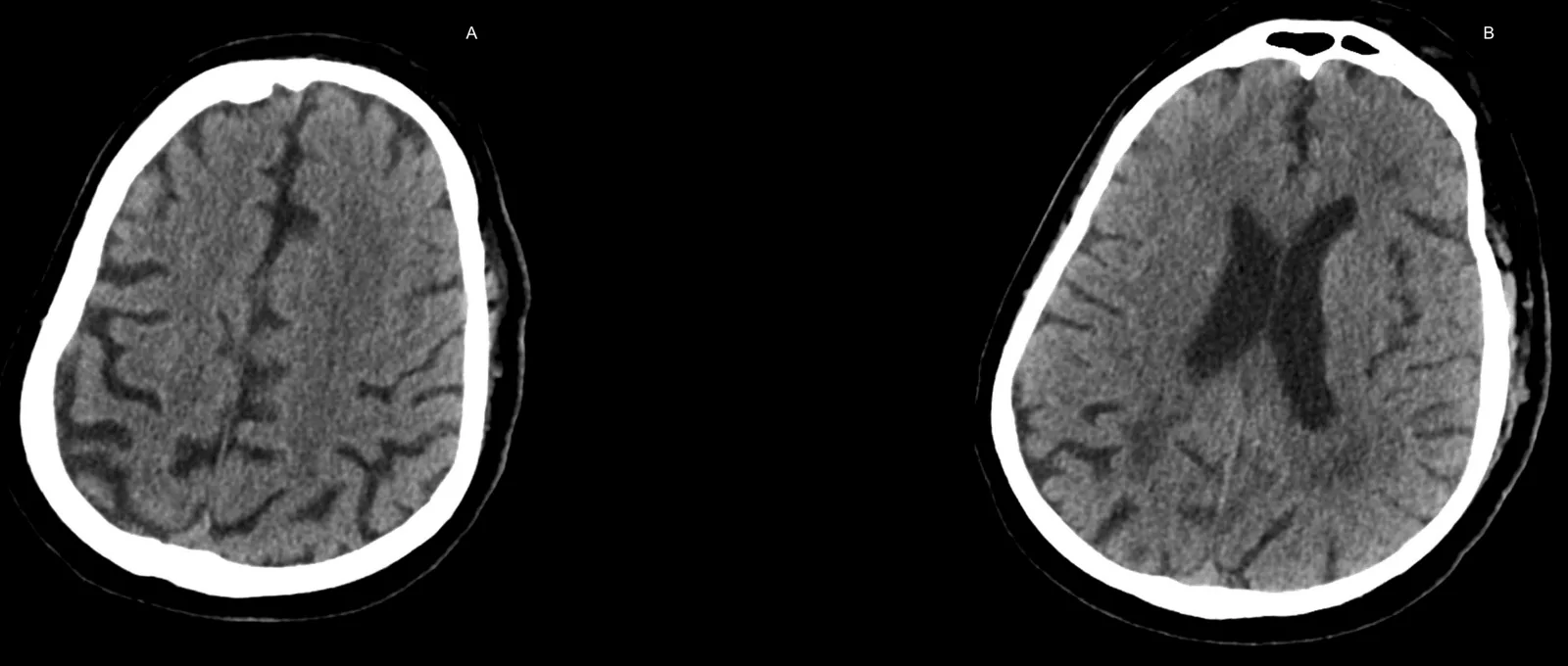
Non-contrast head CT (first admission) A, B: sequential axial non-contrast CT images demonstrating no acute intracranial hemorrhage, mass effect, midline shift, or established large-territory infarct.

A 67-year-old man presented to the emergency department with an acute-onset altered mental status. His past medical history was significant for a prior cerebrovascular accident (CVA) with residual left-sided neurologic deficits; atrial flutter on apixaban (Eliquis); heart failure with reduced ejection fraction (HFrEF); severe aortic stenosis status post bioprosthetic valve placement; chronic kidney disease stage IIIb; uncontrolled type 2 diabetes mellitus; chronic bilateral lower extremity wounds; and obstructive sleep apnea (OSA). The patient met systemic inflammatory response syndrome (SIRS) criteria [5] on initial presentation with tachycardia, tachypnea, and leukocytosis on complete blood count. Blood cultures were obtained in the emergency department, and the patient was initiated on empiric broad-spectrum antimicrobial therapy with intravenous vancomycin (2 g loading dose, then 1,750 mg (≈15 mg/kg) every 18 hours, renally adjusted) and cefepime (2 g every eight hours), in addition to resuscitative intravenous fluids. Given his acute altered mental status, a non-contrast CT of the head was obtained to evaluate for acute intracranial pathology, which returned negative for any acute intracranial process (Figure 1).

**Figure 2:**
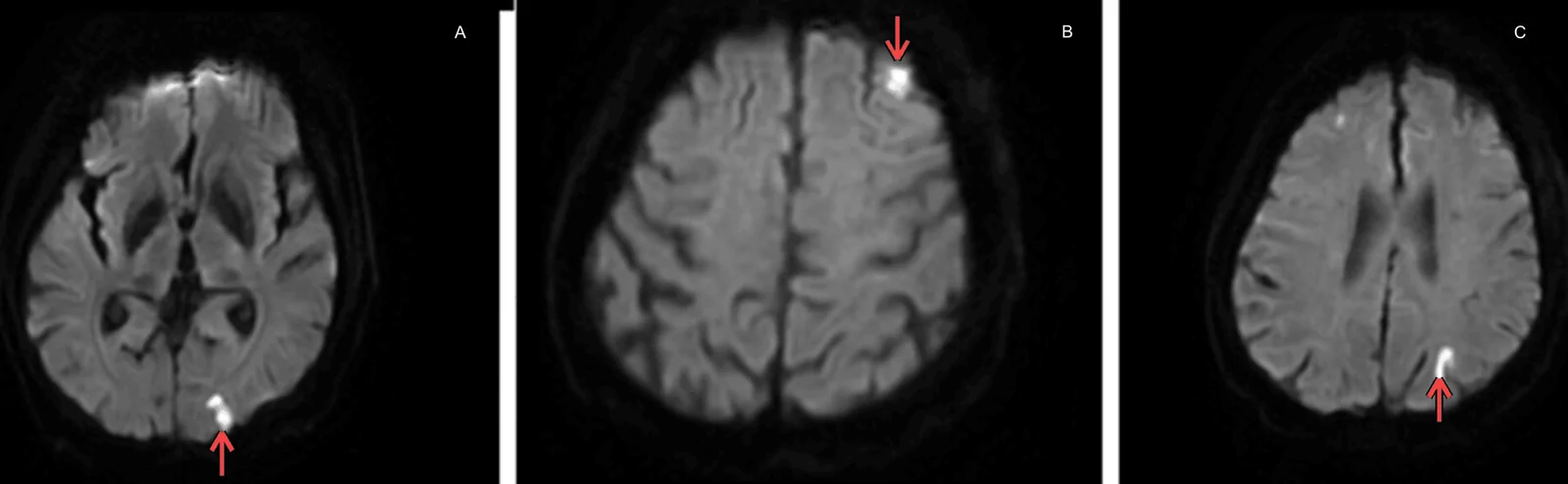
Brain MRI (first admission), diffusion-weighted imaging A-C: arrows indicate scattered bilateral foci of restricted diffusion consistent with acute-to-subacute thromboembolic infarcts with a multifocal, multi-territory pattern that indicates an embolic rather than a single large-vessel source.

On hospital day three, blood cultures grew *Enterococcus faecalis* and *Pseudomonas aeruginosa*; cultures from the chronic lower-extremity wounds grew the same two organisms, confirming the wounds as the bacteremic portal of entry. Bacteremia persisted at 72 hours despite broad antipseudomonal coverage. Vancomycin was discontinued for pre-renal acute kidney injury in the setting of septic shock; given nasal methicillin-resistant *Staphylococcus aureus *(MRSA) colonization, linezolid (600 mg every 12 hours) was started while cefepime was continued, and tobramycin (680 mg every other day) was later added for synergy with IE under active consideration. Concurrently, brain MRI demonstrated multiple scattered bilateral acute and subacute infarcts (Figure 2). In the context of atrial flutter and a bioprosthetic valve, this raised concern for either anticoagulation failure on apixaban or septic embolism from an endovascular source.

**Figure 3:**
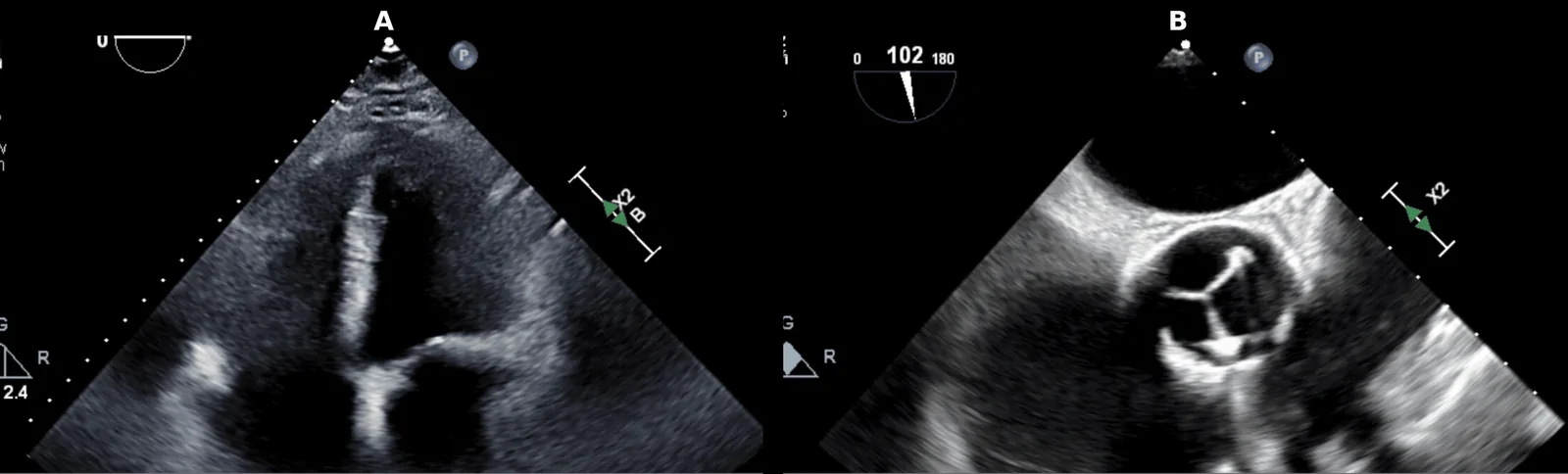
Imaging findings (first admission) Transthoracic (A) and transesophageal (B) echocardiography, demonstrating the bioprosthetic aortic valve without demonstrable vegetation, abscess, or periannular complication.

Therapeutic heparin infusion was initiated. Cardiology was consulted and attributed the embolic events to apixaban failure in the setting of systemic infection and subtherapeutic anticoagulation. Infectious disease (ID) was also consulted and raised concern for hematogenous seeding of the bioprosthetic valve with valvular vegetation as the embolic source. TTE was performed and demonstrated no echocardiographic evidence of valvular vegetations or atrial thrombi. TEE was subsequently performed, which also failed to demonstrate vegetations on the bioprosthetic valve or periannular complications (Figure 3).

On hospital day four, a rapid response was activated for profound hypotension and tachycardia, and the patient was transferred to the medical intensive care unit for vasopressor-dependent septic shock. He stabilized, was weaned off pressors, and was downgraded. Apixaban was discontinued, and he was transitioned from heparin to warfarin. A third set of blood cultures demonstrated clearance of bacteremia, and he was discharged with a peripherally inserted central catheter (PICC) to complete a six-week course of intravenous cefepime (2 g every eight hours) with two weeks of tobramycin.

Rationale for Not Pursuing Source Control at This Stage

At this first admission, IE had not yet been established: it was a first bacteremic episode, echocardiography was negative, the embolic events were initially attributed to anticoagulation failure, and the bacteremia cleared with antimicrobial therapy. Surgical source control was therefore not pursued at that time; it was the recurrence during active therapy that reframed the bioprosthetic valve as the persistent infectious nidus.

Second Admission (March)

**Figure 4:**
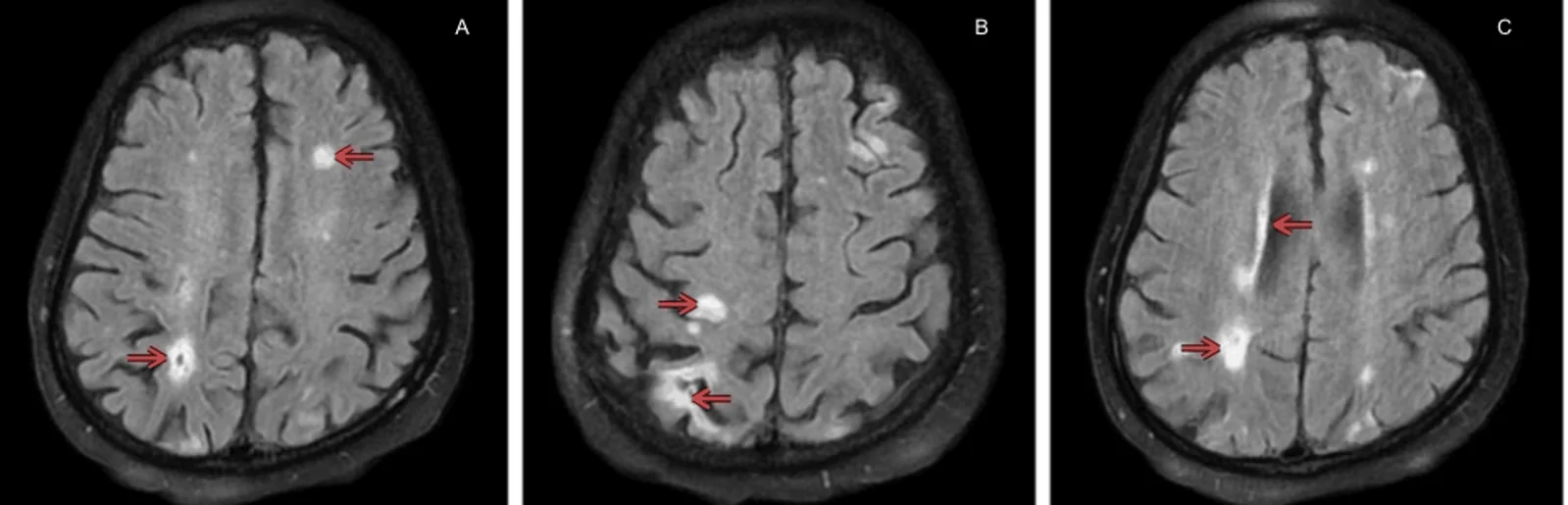
Brain MRI (second admission), fluid-attenuated inversion recovery imaging A-C: arrows indicate new bilateral foci consistent with interval recurrent thromboembolic infarction, evidence of ongoing embolization despite treatment, strongly implicating a persistent embolic source.

Approximately six weeks after discharge, before completing his outpatient regimen, the patient was readmitted with recurrent altered mental status. He was again bacteremic with the identical organism pair, *Pseudomonas aeruginosa *and *Enterococcus faecalis*, and brain MRI demonstrated new bilateral acute thromboembolic infarcts (Figure 4), representing a second episode of polymicrobial bacteremia with the same organisms, in a patient with a prosthetic valve, occurring during active antibiotic therapy.

**Figure 5:**
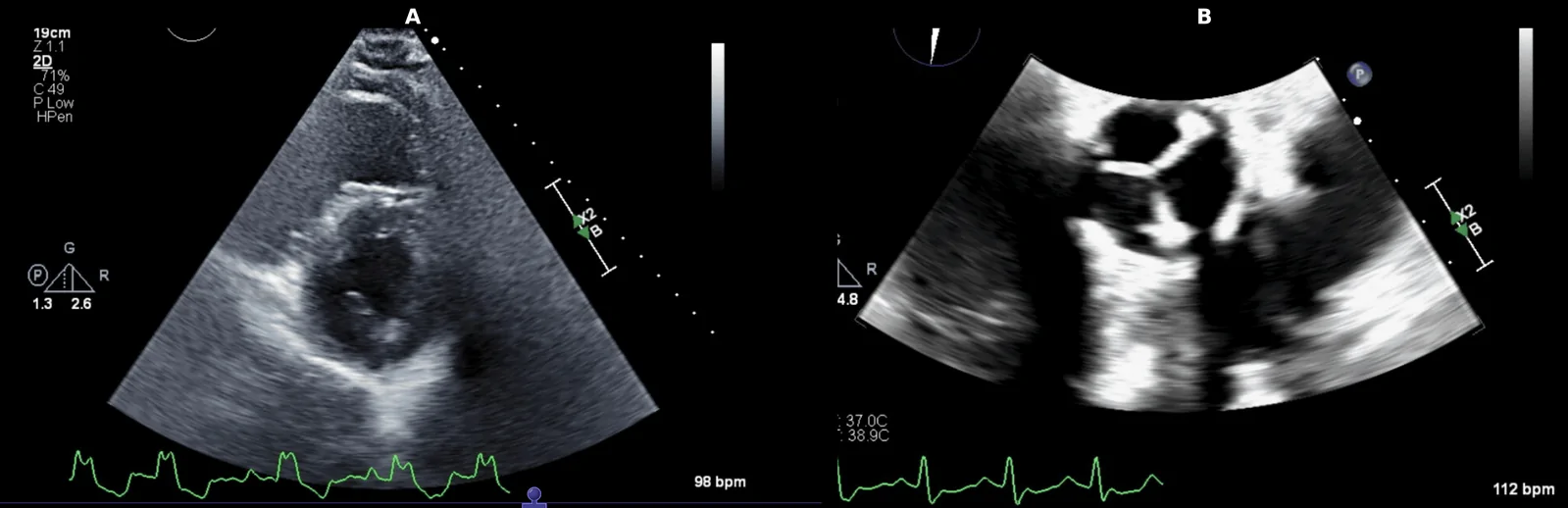
Imaging findings (second admission) Repeat transthoracic (A) and transesophageal (B) echocardiography, again without echocardiographic evidence of vegetation or periannular involvement.

Repeat TTE and TEE again demonstrated no vegetations (Figure 5). Despite this, the clinical picture was compelling: a prosthetic valve, recurrent bacteremia with organisms known to cause IE, recurrent septic cerebral arterial emboli, and documented failure of prolonged targeted therapy to achieve a durable cure. This constellation fulfilled the modified Duke criteria for definite IE (one major criterion with persistently positive blood cultures drawn >12 hours apart plus three minor criteria: a predisposing prosthetic valve, vascular/embolic phenomena, and microbiological evidence not meeting a major criterion) on both admissions (Table 2).

**Table 2:** Application of modified Duke criteria for infective endocarditis IE: infective endocarditis; TTE: transthoracic echocardiography; TEE: transesophageal echocardiography; MRI: magnetic resonance imaging

Criterion	Duke definition (modified)	Present in this case
Major #1: Blood Cultures	Typical organism from >=2 separate blood cultures (*Enterococcus* species qualifies only in the absence of a primary focus) OR persistently positive blood cultures >12 hours apart	YES: persistently positive blood cultures drawn >12 hours apart, with bacteremia beyond 72 hours on both admissions
Major #2: Endocardial Involvement	Vegetation, abscess, or new prosthetic dehiscence on echocardiography; new valvular regurgitation	NO: TTE and TEE negative for vegetations on both admissions
Minor #1: Predisposing Condition	Known predisposing cardiac condition or IV drug use	YES: Bioprosthetic valve
Minor #2: Fever	Temperature >38°C	NO: Afebrile on presentation
Minor #3: Vascular Phenomena	Arterial emboli, septic pulmonary infarcts, mycotic aneurysm, intracranial hemorrhage, Janeway lesions	YES: Multiple bilateral thromboembolic cerebral infarcts on MRI (both admissions)
Minor #4: Microbiologic	Positive blood cultures not meeting major criteria, or serologic evidence of active infection	YES: *Pseudomonas aeruginosa* (not a typical IE organism per major criterion, but supports diagnosis in prosthetic valve context)
Overall Classification	Definite IE = 2 Major; 1 Major + 3 Minor; OR 5 Minor criteria	Definite IE met: 1 Major (blood cultures) + 3 Minor criteria (predisposing condition, nontypical organism bacteremia, vascular emboli)

Vascular surgery was consulted, and a detailed discussion was held with the patient and his family regarding the risks and potential benefits of valve explantation and replacement as definitive source control. Given the complexity of the planned surgical intervention, the patient and family elected to pursue evaluation and operative management at a tertiary care facility. During this second hospitalization, the patient was treated with intravenous meropenem (500 mg every six hours) and linezolid (600 mg every 12 hours); therapy was subsequently de-escalated to piperacillin-tazobactam (4.5 g every eight hours), tobramycin, and oral ciprofloxacin (750 mg every 12 hours) based on susceptibilities. His altered mental status again improved, and blood cultures cleared. He was stabilized, and arrangements were coordinated for transfer to a higher-level center for surgical consideration.

## Discussion

This case of recurrent polymicrobial bacteremia with septic thromboembolic stroke and a bioprosthetic valve as the probable nidus, despite serial negative TTE and TEE on two separate admissions, illustrates a scenario in which clinical judgment, not any single imaging result, must govern diagnosis and management. Notably, although concordant wound cultures identified the lower-extremity wounds as the portal of entry, recurrent arterial embolization to the brain requires a left-sided endovascular source; in a patient with a prosthetic aortic valve and relapsing bacteremia, the valve is the most plausible seeded nidus, and wound bacteremia alone does not account for the cerebral arterial emboli.

Limitations of echocardiography in prosthetic valve endocarditis

TEE sensitivity for native-valve vegetations approaches 87-100% but falls to roughly 82-96% in prosthetic valves, with clinically meaningful false-negative rates in high-pretest-probability scenarios [3]. Several factors reduce yield: acoustic shadowing and reverberation from prosthetic rings and sewing cuffs obscure the leaflet apparatus; vegetations on bioprosthetic leaflets may be small, flat, or non-oscillating; spatial resolution is limited for structures smaller than 2-3 mm; and early vegetations may lack echogenic mass characteristics [3]. In this patient, imaging of the bioprosthesis was inherently degraded by shadowing of the sewing ring and leaflets, and it is clinically plausible that vegetations were present on both admissions but below the threshold of detection [3]. Advanced imaging modalities, including fluorodeoxyglucose (FDG)-PET/CT, cardiac CT angiography, and cardiac MRI, have emerged as important adjuncts when echocardiography is non-diagnostic [6,7], and should be considered early when clinical suspicion remains high.

The modified Duke criteria and clinical diagnosis

The modified Duke criteria, developed by Li and colleagues in 2000 [4], building on the original 1994 criteria [8] and since updated as the 2023 Duke-ISCVID criteria [9] and endorsed by the American Heart Association (AHA) [10] and European Society of Cardiology (ESC) [11], stratify patients into definite, possible, or rejected IE using major and minor criteria drawn from microbiological, echocardiographic, and clinical data.

The major microbiological criterion is met either by a typical IE organism from ≥2 separate cultures or by persistently positive cultures drawn >12 hours apart. We based the major criterion on the persistence of bacteremia (>72 hours, cultures >12 hours apart). *P. aeruginosa*, not a typical IE organism, contributed a minor criterion. The diagnosis, therefore, rested on one major plus three minor criteria: a prosthetic valve, embolic vascular phenomena, and non-major microbiological evidence satisfying definite IE on both admissions. Clinicians should be comfortable making this diagnosis when echocardiographic major criteria are absent; the criteria were designed for exactly this circumstance.

The 2023 Duke-ISCVID revision is particularly relevant to PVE. It broadened the major criteria to incorporate 18F-FDG-PET/CT and cardiac CT findings alongside echocardiography, expanded the lists of typical microorganisms and predisposing conditions, and thereby improved sensitivity in prosthetic valve disease, where echocardiography most often falls short [9]. Under either framework, this patient met criteria for definite IE; the advanced imaging that constitutes a new major criterion under the 2023 system was not obtained here and, had it been, might have provided direct endocardial evidence to complement the clinical diagnosis.

Refractory bacteremia and the imperative of source control

Persistent or recurrent bacteremia (failure to clear within 72 hours of appropriate therapy, or recurrence after apparent clearance) is a major red flag demanding evaluation for a removable nidus [12]. Antimicrobials alone are frequently insufficient to sterilize biofilm-embedded organisms on prosthetic material, and a durable cure requires physical removal or debridement of the infected focus [13]. *Pseudomonas aeruginosa* is notoriously difficult to eradicate from prosthetic surfaces owing to robust biofilm formation, efflux-pump-mediated resistance, and chronic low-grade infection refractory to prolonged antibiotics [13]; *Enterococcus* species similarly persist in biofilm and tolerate cell-wall-active agents [10]. Two biofilm-forming organisms in a patient with a prosthetic valve who could not achieve durable clearance constitute a compelling indication for surgical source control.

Current AHA and ESC guidelines identify indications for urgent surgery, including heart failure from valvular dysfunction; uncontrolled infection (persistent fever or bacteremia beyond five to seven days despite appropriate therapy or abscess, fistula, or enlarging vegetation); and prevention of embolism in patients with high-risk vegetations and prior embolic events [10,11]. This patient met at least two conditions: uncontrolled infection (persistent and recurrent bacteremia) and recurrent embolic events, which, independent of echocardiographic findings, justified referral for source control. Anchoring on a negative echocardiogram is a well-described cognitive error that can delay escalation and surgical referral in suspected PVE [14]; here, reasoning across the totality of data appropriately took precedence.

Diagnostic algorithm when echocardiography is non-diagnostic

When initial echocardiography is negative but suspicion for PVE remains high, a stepwise approach is essential. Repeat echocardiography at five to seven days may detect vegetations that have enlarged and become more echogenic [11]. 18F-FDG-PET/CT is a valuable adjunct in PVE (sensitivity ≈73%, specificity ≈80%), and abnormal peri-prosthetic activity is now a minor and, under the 2023 revision, a major diagnostic criterion [7,9]. Cardiac CT angiography complements this by detecting periannular complications such as abscess or pseudoaneurysm not seen on echocardiography [6]. Early activation of a multidisciplinary endocarditis team with cardiology, ID and cardiac or vascular surgery is associated with improved survival and guideline-concordant care and should occur early in any suspected PVE with persistent bacteremia [15].

Anticoagulation considerations

Anticoagulation in PVE complicated by embolic stroke requires individualization. This patient was on apixaban for atrial flutter; after bilateral thromboembolic strokes and initiation of therapeutic heparin, apixaban was discontinued, and he was transitioned to warfarin. AHA guidance suggests that, in PVE with embolic ischemic stroke without hemorrhagic transformation, continuation of anticoagulation may be considered when the indication is strong, though the risk of hemorrhagic transformation of septic emboli must be weighed [10]. The transition to warfarin was reasonable, affording more titratable anticoagulation aligned with guideline-preferred management for prosthetic-valve-related anticoagulation during active infection.

Limitations

This report has limitations. Advanced imaging (18F-FDG-PET/CT and cardiac CT) was not obtained and could have provided direct endocardial or peri-prosthetic evidence. Pathologic confirmation was unavailable because the patient was transferred before explantation, so the bioprosthetic valve remained the most probable rather than proven nidus. Leading alternative diagnoses were considered, such as cardioembolism from atrial flutter with anticoagulation failure and nonbacterial thrombotic (marantic) endocarditis, but the recurrent polymicrobial bacteremia with biofilm-forming organisms, the prosthetic substrate, embolization during therapeutic anticoagulation, and the confirmed concordant portal of entry made IE the most coherent unifying diagnosis. As a single case, its conclusions are illustrative rather than definitive.

## Conclusions

Recurrent polymicrobial bacteremia with bilateral thromboembolic strokes in a patient with a bioprosthetic valve and two consecutive negative echocardiograms illustrates core principles of IE management. Echocardiography, while essential, carries a meaningful false-negative rate in PVE, and a negative study must be interpreted within the full clinical context. The modified Duke criteria and their 2023 Duke-ISCVID update enable a diagnosis of possible or definite IE when echocardiographic confirmation is lacking. Persistent or recurrent bacteremia with organisms known to cause endocarditis, in the setting of a prosthetic valve and ongoing septic emboli, is a compelling indication for surgical source control irrespective of imaging. Looking forward, when suspicion for PVE persists despite negative echocardiography, clinicians should move early to multimodal imaging (18F-FDG-PET/CT, cardiac CT) and early endocarditis team and surgical referral rather than repeating antibiotic courses in culture-positive, echocardiography-negative PVE. No single negative imaging result should deter definitive treatment.

## References

[1] Li M, Kim JB, Sastry BK, Chen M (2024). Infective endocarditis. Lancet.

[2] Otto CM, Nishimura RA, Bonow RO (2021). ACC/AHA guideline for the management of patients with valvular heart disease: a report of the American College of Cardiology/American Heart Association Joint Committee on clinical practice guidelines. J Am Coll Cardiol.

[3] Cuervo G, Quintana E, Regueiro A, Perissinotti A, Vidal B, Miro JM, Baddour LM (2024). The clinical challenge of prosthetic valve endocarditis: JACC Focus Seminar 3/4. J Am Coll Cardiol.

[4] Li JS, Sexton DJ, Mick N (2000). Proposed modifications to the Duke criteria for the diagnosis of infective endocarditis. Clin Infect Dis.

[5] Bone RC (1995). Sepsis, sepsis syndrome, and the systemic inflammatory response syndrome (SIRS). Gulliver in Laputa. JAMA.

[6] Mgbojikwe N, Jones SR, Leucker TM, Brotman DJ (2019). Infective endocarditis: beyond the usual tests. Cleve Clin J Med.

[7] Primus CP, Clay TA, McCue MS (2022). 18F-FDG PET/CT improves diagnostic certainty in native and prosthetic valve infective endocarditis over the modified Duke criteria. J Nucl Cardiol.

[8] Durack DT, Lukes AS, Bright DK (1994). New criteria for diagnosis of infective endocarditis: utilization of specific echocardiographic findings. Am J Med.

[9] Fowler VG, Durack DT, Selton-Suty C (2023). The 2023 Duke-International Society for Cardiovascular Infectious Diseases criteria for infective endocarditis: updating the modified Duke criteria. Clin Infect Dis.

[10] Baddour LM, Wilson WR, Bayer AS (2015). Infective endocarditis in adults: diagnosis, antimicrobial therapy, and management of complications: a scientific statement for healthcare professionals from the American Heart Association. Circulation.

[11] Delgado V, Ajmone Marsan N, de Waha S (2023). 2023 ESC guidelines for the management of endocarditis. Eur Heart J.

[12] Grapsa J, Blauth C, Chandrashekhar YS, Prendergast B, Erb B Jr, Mack M, Fuster V (2022). Staphylococcus aureus infective endocarditis: JACC Patient Pathways. J Am Coll Cardiol.

[13] Ciofu O, Tolker-Nielsen T (2019). Tolerance and resistance of Pseudomonas aeruginosa biofilms to antimicrobial agents—how P. aeruginosa can escape antibiotics. Front Microbiol.

[14] Croskerry P (2003). The importance of cognitive errors in diagnosis and strategies to minimize them. Acad Med.

[15] Roy AS, Hagh-Doust H, Abdul Azim A (2023). Multidisciplinary teams for the management of infective endocarditis: a systematic review and meta-analysis. Open Forum Infect Dis.

